# Carbapenem-resistant enterobacteriacae: a challenge for early detection and infection control

**DOI:** 10.1186/1753-6561-5-S6-P142

**Published:** 2011-06-29

**Authors:** C Fankhauser, A Cherkaoui, G Renzi, M Abbas, J Schrenzel, D Pittet, S Harbarth

**Affiliations:** 1 University of Geneva Hospitals, Geneva, Switzerland

## Introduction / objectives

In 2009, in response to the threat of emerging carbapenem-resistant Enterobacteriaceae, an alert system was introduced at Geneva University Hospitals.

## Methods

The alert system detected patients harboring carbapenemase-producing strains as KPC or non-KPC. Referrals from other hospitals were screened on admission for the presence of multiresistant organisms, and put under contact control precautions if positive.

## Results

Between October 2009 - January 2010, we identified 1 imported case of KPC (origin, Southern Italy) and 3 cases of NDM-1 producing Enterobacteriaceae, transferred from hospitals in India (1), Pakistan (2) and Serbia/France(3).

Patient 1- on admission digestive carrier of *E. coli bla*_NDM-1._

Patient 2- digestive carrier of *P. mirabilis bla*_NDM-1_ detected after extended hospitalization and antibiotic therapy

Patient 3- hospitalized in Serbia and France, with *K. pneumonia bla*_NDM-1_ urinary tract infection on admission.

All 3 patients were carriers of other multiresistant, Gram-negative bacteria on admission. The NDM-1 molecular identification was made retrospectively in October 2010. Patient 4 was admitted for elective surgery, without prior history of hospitalization. A urine culture yielded *K. pneumoniabla*_KPC-2_. The patient was put under strict contact precautions; but developed a surgical site infection with treatment challenges related to dose finding, availability, toxicity of antibiotics. No secondary cases were found due to early screening and preemptive isolation.

## Conclusion

The threat of carbapenemase-producing strains underlines the need for early detection, implementation of control measures and surveillance, which needs constant updating. The laboratory alert system focused on KPC but ignored initially the NDM-1 threat.

## Disclosure of interest

None declared.

